# The Development and Use of Chatbots in Public Health: Scoping Review

**DOI:** 10.2196/35882

**Published:** 2022-10-05

**Authors:** Lee Wilson, Mariana Marasoiu

**Affiliations:** 1 Centre for Policy Futures University of Queensland St Lucia, Queensland Australia; 2 Department of Computer Science and Technology University of Cambridge Cambridge United Kingdom

**Keywords:** chatbots, conversational agents, public health, evidence, scoping review, health care system, chatbot development, digital health, mental health, health technology, COVID-19, pandemic, chatbot application

## Abstract

**Background:**

Chatbots are computer programs that present a conversation-like interface through which people can access information and services. The COVID-19 pandemic has driven a substantial increase in the use of chatbots to support and complement traditional health care systems. However, despite the uptake in their use, evidence to support the development and deployment of chatbots in public health remains limited. Recent reviews have focused on the use of chatbots during the COVID-19 pandemic and the use of conversational agents in health care more generally. This paper complements this research and addresses a gap in the literature by assessing the breadth and scope of research evidence for the use of chatbots across the domain of public health.

**Objective:**

This scoping review had 3 main objectives: (1) to identify the application domains in public health in which there is the most evidence for the development and use of chatbots; (2) to identify the types of chatbots that are being deployed in these domains; and (3) to ascertain the methods and methodologies by which chatbots are being evaluated in public health applications. This paper explored the implications for future research on the development and deployment of chatbots in public health in light of the analysis of the evidence for their use.

**Methods:**

Following the PRISMA-ScR (Preferred Reporting Items for Systematic Reviews and Meta-Analyses Extension for Scoping Reviews) guidelines for scoping reviews, relevant studies were identified through searches conducted in the MEDLINE, PubMed, Scopus, Cochrane Central Register of Controlled Trials, IEEE Xplore, ACM Digital Library, and Open Grey databases from mid-June to August 2021. Studies were included if they used or evaluated chatbots for the purpose of prevention or intervention and for which the evidence showed a demonstrable health impact.

**Results:**

Of the 1506 studies identified, 32 were included in the review. The results show a substantial increase in the interest of chatbots in the past few years, shortly before the pandemic. Half (16/32, 50%) of the research evaluated chatbots applied to mental health or COVID-19. The studies suggest promise in the application of chatbots, especially to easily automated and repetitive tasks, but overall, the evidence for the efficacy of chatbots for prevention and intervention across all domains is limited at present.

**Conclusions:**

More research is needed to fully understand the effectiveness of using chatbots in public health. Concerns with the clinical, legal, and ethical aspects of the use of chatbots for health care are well founded given the speed with which they have been adopted in practice. Future research on their use should address these concerns through the development of expertise and best practices specific to public health, including a greater focus on user experience.

## Introduction

Sundar Pichai, the chief executive officer of Google, expressed in a recent interview his view that artificial intelligence (AI) will have a more profound impact on humanity than the advent of fire, the internet, or electricity [1]. Although Pichai has a vested interest in propagating visions of AI-enhanced futures, there is no doubting the extent to which advances in computing technology are driving rapid transformations in the ways in which we interact with computing systems, organizations, one another, and the world. A salient feature of this rapidly changing technological landscape is the burgeoning development and use of conversational agents, or “chatbots.”

Chatbots—software programs designed to interact in human-like conversation—are being applied increasingly to many aspects of our daily lives. Made to mimic natural language conversations to facilitate interaction between humans and computers, they are also referred to as “conversational agents,” “dialog assistants,” or “intelligent virtual assistants,” and they can support speech and text conversation. Notable early chatbots include ELIZA (1966;“a mock Rogerian psychotherapist”), PARRY (1972; a chatbot simulating a person with paranoid schizophrenia, developed by a psychiatrist in response to ELIZA), and ALICE (1995; a general conversational chatbot, inspired by ELIZA) [2]. Recent advances in the development and application of chatbot technologies and the rapid uptake of messenger platforms have fueled the explosion in chatbot use and development that has taken place since 2016 [3]. Improvements to natural language processing (NLP; which includes speech recognition, text-to-speech, speech-to-text, natural language understanding, and natural language generation), as well as the emergence and publicity of commercial “virtual assistants” such as Siri, Google Now, Cortana, and Alexa [4] have brought AI into many aspects of our daily lives. Chatbots are now found to be in use in business and e-commerce, customer service and support, financial services, law, education, government, and entertainment and increasingly across many aspects of health service provision [5].

The ongoing COVID-19 pandemic has further driven the rapid uptake and deployment of chatbots [6], many making use of commercial chatbot development platforms such as IBM’s Watson Assistant, Google Dialogflow, Yellow Messenger, and Turn.io to develop custom chatbots to help combat the disease. In the face of the burden placed upon health care systems by the pandemic, chatbots have enabled the automation of services toward addressing the need for physical distancing and helped disseminate information and relieve the pressure on medical services by public health systems around the globe [7,8].

The use of AI for symptom checking and triage at scale has now become the norm throughout much of the world, signaling a move away from human-centered health care [9] in a remarkably short period of time. Recognizing the need to provide guidance in the field, the World Health Organization (WHO) has recently issued a set of guidelines for the ethics and principles of the use of AI in health [10]. WHO has itself made use of chatbots to provide guidance and combat misinformation about COVID-19 through its Health Alert chatbot [11] that communicates in a number of different languages through WhatsApp, Viber, and Facebook messenger, which has reportedly reached over 12 million people [12].

In the light of the huge growth in the deployment of chatbots to support public health provision, there is pressing need for research to help guide their strategic development and application [13]. This paper aimed to help address this deficit. We examined the evidence for the development and use of chatbots in public health to assess the current state of the field, the application domains in which chatbot uptake is the most prolific, and the ways in which chatbots are being evaluated. Reviewing current evidence, we identified some of the gaps in current knowledge and possible next steps for the development and use of chatbots for public health provision. Our research questions are as follows.

1. What does the evidence tell us about the use of chatbots in public health?
2. In which fields of public health have chatbots been used the most frequently?
3. What are the types of chatbots that have been used in public health?
4. How have chatbots in public health been evaluated?
5. What are the potential lessons to be learned from the evidence for the use of chatbots in public health?

## Methods

We carried out a scoping review of studies on the use of chatbots in public health. We followed the PRISMA-ScR (Preferred Reporting Items for Systematic Reviews and Meta-Analyses Extension for Scoping Reviews) guidelines and methodological framework by Arksey and O'Malley [14] for scoping studies and searched the titles and abstracts of studies on the MEDLINE, PubMed, Scopus, Cochrane Central Register of Controlled Trials, IEEE Xplore, ACM Digital Library, and Open Grey databases over a period of 2 weeks in June 2021. Our search terms included “chatbot,” “conversational agent,” and their synonyms and public health, global public health, and related terms (see Multimedia Appendix 1). We chose to broaden our search to include health care and health to gain a broader understanding of the application domains in which chatbots are being used for health-related purposes. The domain categorization was assigned in 3 ways: (1) self-identified by the authors, (2) categorized according to current definitions of public health sectors, and (3) assigned according to the design scope of the chatbot. With regard to mental health, we made a further distinction between chatbots that were specifically designed to provide social support in nondiagnosed patients, defining these as “counseling/support,” and those that were designed to deal with clinical illnesses such as depression, defining these as “mental health.”

The use of AI and digital technologies and the roles in which they are deployed in health tend to blur the boundaries between population and clinical health—that is, chatbots that are used to service individual health needs are often equally as relevant to population-level health in application. In this respect, the synthesis between population-based prevention and clinical care at an individual level [15] becomes particularly relevant. Implicit to digital technologies such as chatbots are the levels of efficiency and scale that open new possibilities for health care provision that can extend individual-level health care at a population level. We have therefore included studies of chatbots designed for the provision of health services to individuals where there is evidence of demonstrable health impacts and, importantly, where they have the potential for scalable efficiencies to support health outcomes at a population level.

Our selection methodology was as follows. One of the authors screened the titles and abstracts of the studies identified through the database search, selecting the studies deemed to match the eligibility criteria. The second author then screened 50% of the same set of identified studies at random to validate the first author’s selection. The papers meeting the criteria for inclusion at the title- and abstract-screening stage were retrieved and reviewed independently by both authors, with subsequent discussion about discrepancies and resolution to end with an agreed upon list of included studies.

Our inclusion criteria were for the studies that used or evaluated chatbots for the purpose of prevention or intervention and for which the evidence showed a demonstrable health impact. We included experimental studies where chatbots were trialed and showed health impacts. We also included feasibility studies for agents that are being rolled out, randomized controlled trials (RCTs) informing the feasibility of conversational agents that have obvious applicability for scalability and potential for population-level interventions, and comparative analyses of in-service chatbots. We chose not to distinguish between embodied conversational agents and text-based agents, including both these modalities, as well as chatbots with cartoon-based interfaces.

**Figure 1:**
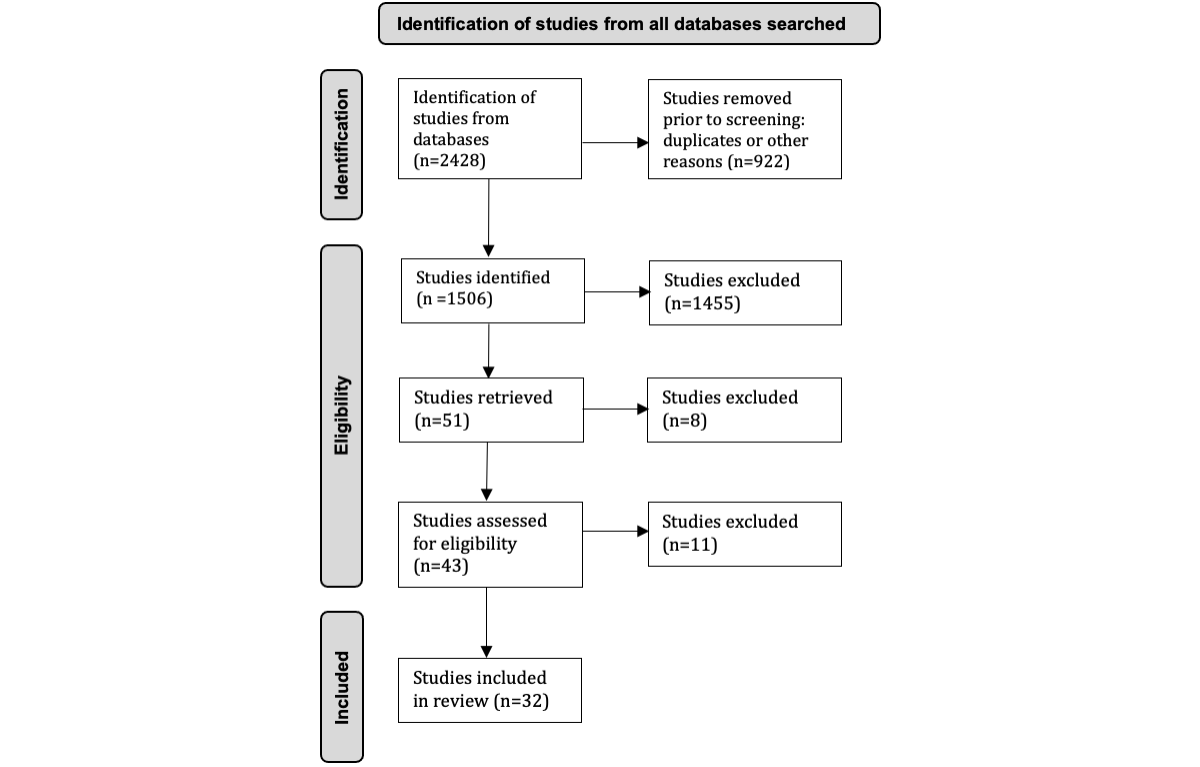
Review selection process.

We excluded thought experiments, design outlines and reflections on systems that have yet to be implemented, descriptions of proposed chatbots and conversational agents, prototypes of system architecture, surveys and predesign analyses, frameworks, commentaries, validation studies, technical papers that introduced agents explaining their architecture and design that have yet to be trialed, and papers exploring perceptions of digital agents or their acceptability or validity among users. We also excluded studies comparing the effect of differences in technical approaches (eg, messaging) and studies that used “Wizard of Oz” protocols—a protocol used to test users’ reactions in which a human responds to users through an interface in which they think they are interacting with a computer. The review selection process is shown in Figure 1.

## Results

### Included Studies

In total, 32 studies met the inclusion criteria. These studies included RCTs (n=12), user analytics (n=8), user experience studies (n=3), an experimental pilot (n=1), a descriptive study (n=1), comparative analyses (n=2), a case control study (n=1), design processes (n=2), and feasibility studies (n=2). These studies were distributed across 11 application domains.

**Figure 2:**
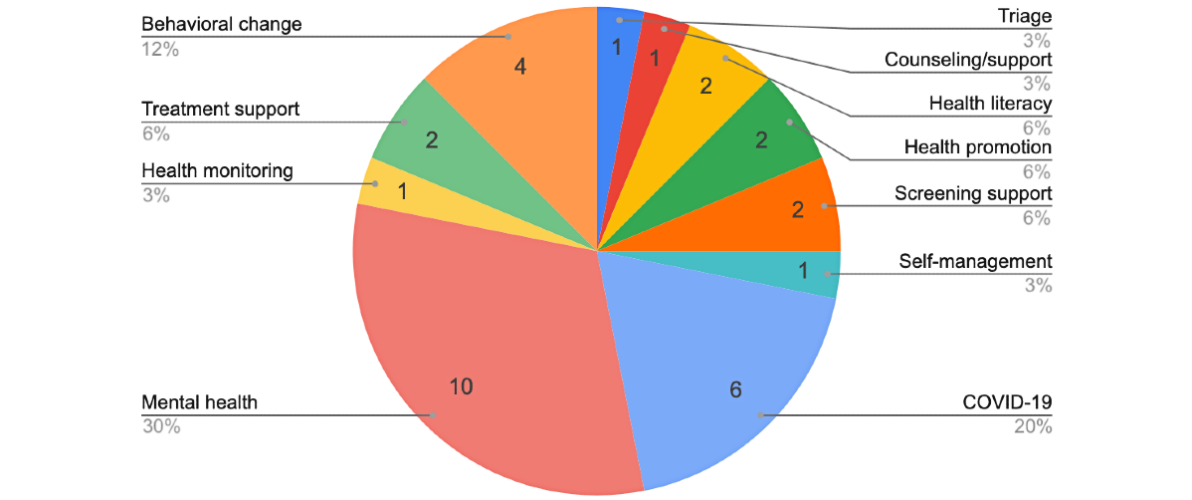
Distribution of included publications across application domains. Mental health research and COVID-19 form the majority of the studies. Due to the small numbers of papers, percentages must be interpreted with caution and only indicate the presence of research in the area rather than an accurate distribution of research.

Mental health and COVID-19 dominated the application domains. This result is possibly an artifact of the maturity of the research that has been conducted in mental health on the use of chatbots and the massive surge in the use of chatbots to help combat COVID-19. The graph in Figure 2 thus reflects the maturity of research in the application domains and the presence of research in these domains rather than the quantity of studies that have been conducted.

### Maturity of Chatbot Research in Public Health Domains

**Figure 3:**
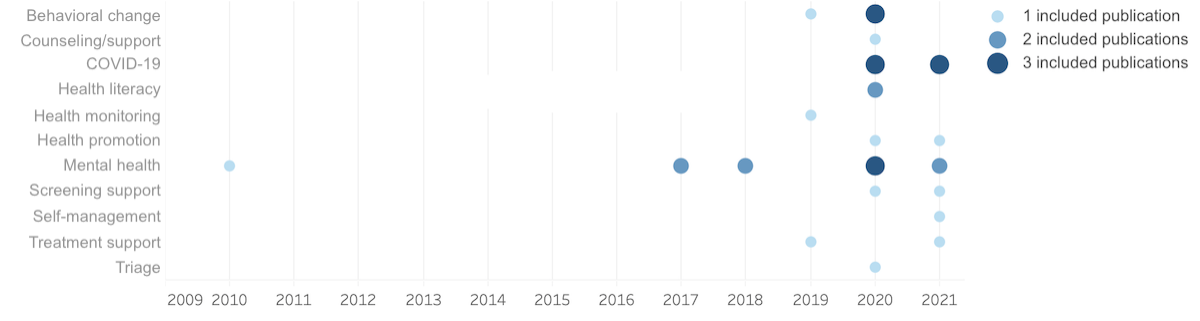
Distribution of included publications across application domains and publication year. Mental health research has a continued interest over time, with COVID-19–related research showing strong recent interest as expected.

The timeline for the studies, illustrated in Figure 3, is not surprising given the huge upsurge of interest in chatbots from 2016 onward. Although health services generally have lagged behind other sectors in the uptake and use of chatbots, there has been greater interest in application domains such as mental health since 2016. This finding may reflect both the degree to which conversational technologies lend themselves to the kinds of interactive methodologies used in mental health and the necessity for greater scrutiny of the methods that are used by health practitioners in field.

Similarly, one can see the rapid response to COVID-19 through the use of chatbots, reflecting both the practical requirements of using chatbots in triage and informational roles and the timeline of the pandemic.

### Chatbot Design

Studies that detailed any user-centered design methodology applied to the development of the chatbot were among the minority (3/32, 9%) [16-18]. Most (22/32, 69%) included papers only described broadly the messaging content available in the chatbot (eg, topics covered) and what functionality was available to the user (eg, daily reminders), but few had a description of the process by which those features and capabilities were decided upon.

One study that stands out is the work of Bonnevie and colleagues [16], who describe the development of *Layla*, a trusted source of information in contraception and sexual health among a population at higher risk of unintended pregnancy. *Layla* was designed and developed through community-based participatory research, where the community that would benefit from the chatbot also had a say in its design. *Layla* demonstrates the potential of AI to empower community-led health interventions. Such approaches also raise important questions about the production of knowledge, a concern that AI more broadly is undergoing a reckoning with [19].

**Figure 4:**
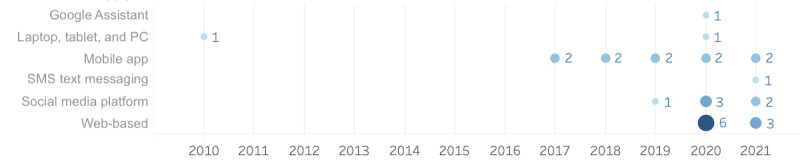
Distribution of chatbot platforms in the included studies. PC: personal computer.

Two-thirds (21/32, 66%) of the chatbots in the included studies were developed on custom-developed platforms on the web [6,16,20-26], for mobile devices [21,27-36], or personal computers [37,38]. A smaller fraction (8/32, 25%) of chatbots were deployed on existing social media platforms such as Facebook Messenger, Telegram, or Slack [39-44]; using SMS text messaging [42,45]; or the Google Assistant platform [18] (see Figure 4).

All the included studies tested textual input chatbots, where the user is asked to type to send a message (free-text input) or select a short phrase from a list (single-choice selection input). Only 4 studies included chatbots that responded in speech [24,25,37,38]; all the other studies contained chatbots that responded in text.

**Figure 5:**
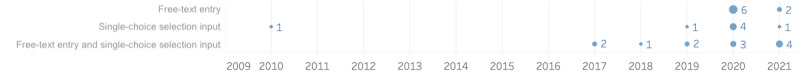
The ways in which users could message the chatbot were either by choosing from a set of predefined options or freely typing text as in a typical messaging app.

The majority (28/32, 88%) of the studies contained very little description of the technical implementation of the chatbot, which made it difficult to classify the chatbots from this perspective. Most (19/32, 59%) of the included papers included screenshots of the user interface. However, some only provided sketches of the interface, and often, the text detailing chatbot capabilities was not congruent with the picture accompanying the text (eg, the chatbot was described as free entry but the screenshot showed a single-choice selection). In such cases, we marked the chatbot as using a combination of input methods (see Figure 5).

Surprisingly, there is no obvious correlation between application domains, chatbot purpose, and mode of communication (see Multimedia Appendix 2 [6,8,9,16-18,20-45]). Some studies did indicate that the use of natural language was not a necessity for a positive conversational user experience, especially for symptom-checking agents that are deployed to automate form filling [8,46]. In another study, however, not being able to converse naturally was seen as a negative aspect of interacting with a chatbot [20].

**Figure 6:**
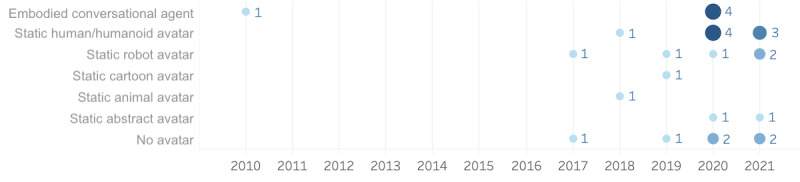
Presentation of the chatbot avatar.

The presentation of the chatbot persona (see Figure 6) was usually presented as a static avatar (n=17). Of these chatbots, 8 were given an anthropomorphic avatar, whether as a photo or drawing (eg, clipart), whereas the rest adopted either a robot, animal, cartoon, or another abstract avatar. Embodied conversational agents (n=5) were only presented as “female” human-like avatars. Of those with no avatars (n=6), this absence was usually due to the platform restriction (eg, WhatsApp or some forms of embedded web chat). The influence of avatar presence and the anthropomorphic appearance of chatbots are still an underresearched area, but we expect it will be of particular importance for future chatbot design in health care.

### Evidence for the Efficacy of Chatbot-Based Health Interventions

The included studies consisted of RCTs (n=12), user analytics (n=8), user experience studies (n=3), an experimental pilot (n=1), a descriptive study (n=1), comparative analyses (n=2), a case control study (n=1), design processes (n=2), and feasibility studies (n=2).

For RCTs, the number of participants varied between 20 to 927, whereas user analytics studies considered data from between 129 and 36,070 users. Overall, the evidence found was positive, showing some beneficial effect, or mixed, showing little or no effect. Evidence was predominantly preliminary and context specific. Most (21/32, 65%) of the included studies established that the chatbots were usable but with some differences in the user experience and that they can provide some positive support across the different health domains.

Moderate positive results were found across several studies in regard to knowledge and skill improvement [20,39], reducing health risks [25], and supporting diet and physical exercise [31], and there is some preliminary evidence of chatbots that support smoking cessation improving the chances of quitting [36].

Studies on the use of chatbots for mental health, in particular anxiety and depression, also seem to show potential, with users reporting positive outcomes on at least some of the measurements taken [33,34,41]. The research suggests that psychotherapy chatbots can act as a supplemental tool as part of the broader psychotherapy process [21] across a broad range of psychotherapeutic methodologies and approaches (see Multimedia Appendix 2 for a summary of chatbot roles).

Chatbots were found to have improved medical service provision by reducing screening times [17] and triaging people with COVID-19 symptoms to direct them toward testing if required. These studies clearly indicate that chatbots were an effective tool for coping with the large numbers of people in the early stages of the COVID-19 pandemic. However, 1 comparative study [22] showed that the number of correctly assessed cases of COVID-19 varied considerably between the 10 web-based symptom checkers, with only 2 chatbots having a good balance between sensitivity (not classifying almost all patients as COVID-19–positive) and specificity (not classifying all patients as COVID-19–negative). Overall, this result suggests that although chatbots can achieve useful scalability properties (handling many cases), accuracy is of active concern, and their deployment needs to be evidence-based [23].

The evidence for the use of chatbots to support behavior change is mixed. One study found that any effect was limited to users who were already contemplating such change [24], and another study provided preliminary evidence for a health coach in older adults [31]. Another study reported finding no significant effect on supporting problem gamblers despite high completion rates [40].

Mixed findings were also reported regarding adherence. One study found that there was no effect on adherence to a blood pressure–monitoring schedule [39], whereas another reported a positive improvement medication adherence [35].

Research on the use of chatbots in public health service provision is at an early stage. Although preliminary results do indicate positive effects in a number of application domains, reported findings are for the most part mixed. Moreover, varying user engagement with the chatbots (though not necessarily correlated with the effect [36]), the size of the study, and the demographic characteristics of the target population (eg, some groups of people were more likely to have a better experience using the chatbot [18]) are some of the few variables that might affect the efficacy of an intervention.

### Evaluation of Chatbot Design

The majority (26/32, 81%) of the studies used quantitative methods and methodologies for the evaluation of chatbot design and their impact in relation to health outcomes. For the most part, qualitative methods were used to examine the acceptability of chatbots to patients and their self-reported experience in using them alongside other quantitative usability metrics [45]. User experience and usability evaluation consisted of structured questionnaires and surveys, usually with a few open-ended questions (n=11), and just 1 study used a focus group (n=1).

By far the most prevalent means of assessing health impacts of chatbot-led interventions were RCTs (n=12). Studies that focused on the effectiveness of chatbots with regard to an assigned task, such as triage and symptom checking, lent themselves more easily to evaluation through user analytics (n=8). There was, however, limited evaluation of user experience (n=3), and chatbot development was rarely design-led—although there were notable exceptions, with 1 study identifying user principles in development [17]; 1 study following a human-centered design process with young adults treated for cancer [43]; and another using community based, participatory research to develop a chatbot [16]. A further limitation noted in the evaluation of widely deployed chatbots is that the data collected by the chatbot for further analysis do not hold personally identifiable information, so it is not possible to know if the targeted population are the actual users [16].

No included studies reported direct observation (in the laboratory or in situ; eg, ethnography) or in-depth interviews as evaluation methods. Given the recognized need for observational study in chatbots deployed for public health [28] and the current widespread use of observational and participatory methodologies in human-computer interaction (HCI) [47], there is an impetus for future chatbot research to rely on such methodologies if their development is to best support their users.

## Discussion

### Principal Findings

Although research on the use of chatbots in public health is at an early stage, developments in technology and the exigencies of combatting COVID-19 have contributed to the huge upswing in their use, most notably in triage roles. Studies on the use of chatbots for mental health, in particular depression, also seem to show potential, with users reporting positive outcomes [33,34,41]. Impetus for the research on the therapeutic use of chatbots in mental health, while still predominantly experimental, predates the COVID-19 pandemic. However, the field of chatbot research is in its infancy, and the evidence for the efficacy of chatbots for prevention and intervention across all domains is at present limited.

Notably, people seem more likely to share sensitive information in conversation with chatbots than with another person [20]. Speaking with a chatbot and not a person is perceived in some cases to be a positive experience as chatbots are seen to be less “judgmental” [48]. Human-like interaction with chatbots seems to have a positive contribution to supporting health and well-being [27] and countering the effects of social exclusion through the provision of companionship and support [49]. However, in other domains of use, concerns over the accuracy of AI symptom checkers [22] framed the relationships with chatbot interfaces. The trustworthiness and accuracy of information were factors in people abandoning consultations with diagnostic chatbots [28], and there is a recognized need for clinical supervision of the AI algorithms [9].

Although the COVID-19 pandemic has driven the use of chatbots in public health, of concern is the degree to which governments have accessed information under the rubric of security in the fight against the disease. For example, in South Korea, the implementation of integrated technological responses, including personalized communication chatbots and the use of personal data gathered for contact tracing [50], uses AI in a way that transgresses what many would argue are fundamental human rights to privacy. The sharing of health data gathered through symptom checking for COVID-19 by commercial entities and government agencies presents a further challenge for data privacy laws and jurisdictional boundaries [51].

The evidence cited in most of the included studies either measured the effect of the intervention or surface and self-reported user satisfaction. There was little qualitative experimental evidence that would offer more substantive understanding of human-chatbot interactions, such as from participant observations or in-depth interviews. In this respect, we should remember that chatbots are complex systems, and chatbot deployment in public health is a technology design activity (the design of the platform, the communication modality, and content), as much as it is a medical intervention (the design of the intervention and setting up measures for its effectiveness). As an interdisciplinary subject of study for both HCI and public health research, studies must meet the standards of both fields, which are at times contradictory [52]. Methods developed for the evaluation of pharmacological interventions such as RCTs, which were designed to assess the effectiveness of an intervention, are known in HCI and related fields [53] to be limited in the insights they provide toward better design.

Studies in the existing research often do not provide sufficient information about the design of the chatbot being tested to be reproducible, including by RCT standards, as the chatbot description is not sufficient for an equivalent chatbot to be implemented. There are further confounding factors in the intervention design that are not directly chatbot related (eg, daily notifications for inputting mood data) or include aspects such as the chatbot’s programmed personality that affect people differently [33]. As an emerging field of research, the future implications of human interactions with AI and chatbot interfaces is unpredictable, and there is a need for standardized reporting, study design [54,55], and evaluation [56].

Few of the included studies discussed how they handled safeguarding issues, even if only at the design stage. Of those that did, the studies mentioned that they could not provide a person to support the chatbot (ie, conversations with the chatbot are not monitored by a person), so the chatbot was programmed to message the user to contact official health authorities if they had an issue (eg, directing the user to call 911). This methodology is a particular concern when chatbots are used at scale or in sensitive situations such as mental health. In this respect, chatbots may be best suited as supplements to be used alongside existing medical practice rather than as replacements [21,33].

### Implications for Future Research

Although the use of NLP is a new territory in the health domain [47], it is a well-studied area in computer science and HCI. When developing and deploying new technological interventions, one must take care to identify the ways in which these interventions might replicate or amplify existing inequities, such as access to language proficiency, technology literacy, smartphone technology, mobile data, and even electricity [9]. Human-centered design processes used in HCI and computer science, particularly those that engage the target user throughout the design process such as participatory design, co-design, and participatory action research, could be useful methods for addressing existing inequities from the beginning [57].

Most of the included papers contained screenshots of the chatbots. However, some of these were sketches of the interface rather than the final user interface, and most of the screenshots had insufficient description as to what the capabilities were. Although the technical descriptions of chatbots might constitute separate papers in their own right, these descriptions were outside the scope for our focus on evidence in public health. However, a previously published scoping review [58], focusing on the technical aspects of chatbots’ implementation for medical use, distinguished between text-understanding modality (eg, pattern matching, machine learning, fixed input, and hybrid), data management (medical knowledge database, user information database, and conversation scripts), and text generation (fixed output and machine learning). A further scoping study would be useful in updating the distribution of the technical strategies being used for COVID-19–related chatbots.

Future research on chatbots would benefit from including more details as to how the chatbot is implemented and what type of NLP it uses and cross-referencing the equivalent technical paper describing the system implementation and technical contribution, if it is available.

More broadly, in a rapidly developing technological field in which there is substantial investment from industry actors, there is a need for better reporting frameworks detailing the technologies and methods used for chatbot development. Similarly, given the huge range of chatbot deployments across a wide variety of public health domains, there is a need for standards of comparative criteria to facilitate a better evaluation and validation of these agents and the methods and approaches that they use to improving health and well-being. Finally, there is a need to understand and anticipate the ways in which these technologies might go wrong and ensure that adequate safeguarding frameworks are in place to protect and give voice to the users of these technologies.

### Limitations

Given the immaturity of the research on chatbots, the huge investment in their development and use for health, and the dynamic nature of AI and HCI, our study does not capture the abundance of chatbots, commercial and otherwise, that have been developed across of the domains of public health application. There is a substantial lag between the production of academic knowledge on chatbot design and health impacts and the progression of the field.

### Conclusions

Research on the recent advances in AI that have allowed conversational agents more realistic interactions with humans is still in its infancy in the public health domain. Studies show potential, especially for easily automated and repetitive tasks, but at the same time, concerns with the clinical, legal, and ethical aspects of the use of conversational agents for health care are well founded given the speed with which they have been adopted in practice. There is still little evidence in the form of clinical trials and in-depth qualitative studies to support widespread chatbot use, which are particularly necessary in domains as sensitive as mental health. Most of the chatbots used in supporting areas such as counseling and therapeutic services are still experimental or in trial as pilots and prototypes. Where there is evidence, it is usually mixed or promising, but there is substantial variability in the effectiveness of the chatbots. This finding may in part be due to the large variability in chatbot design (such as differences in content, features, and appearance) but also the large variability in the users’ response to engaging with a chatbot.

There is no doubting the extent to which the use of AI, including chatbots, will continue to grow in public health. The ethical dilemmas this growth presents are considerable, and we would do well to be wary of the enchantment of new technologies [59]. Of paramount concern is the need to understand where we can use automation over other technologies that connect humans to humans (eg, machine assistance instead of machine intelligence) and what are the situations in which a conversation with a computer that simulates another person is indeed serving the needs of the person. For example, the recently published WHO Guidance on the Ethics and Governance of AI in Health [10] is a big step toward achieving these goals and developing a human rights framework around the use of AI. However, as Privacy International commented in a review of the WHO guidelines, the guidelines do not go far enough in challenging the assumption that the use of AI will inherently lead to better outcomes [60]. Digital innovation in public health should ideally be informed by research that measures the impact that technologies such as chatbots may have in health interventions, provides insight into user experience, and works to ensure the safety of and promote the well-being of users.

## Appendix

Multimedia Appendix 1 Search terms.

Multimedia Appendix 2 Chatbot application domain, purpose, interaction type, and findings summary.

## Abbreviations

AI: artificial intelligence
HCI: human-computer interaction
NLP: natural language processing
PRISMA-ScR: Preferred Reporting Items for Systematic Reviews and Meta-Analyses Extension for Scoping Reviews
RCT: randomized controlled trial
WHO: World Health Organization
