# Supplementary material for: The Development and Use of Chatbots in Public Health: Scoping Review
Source: JMIR Hum Factors. 2022 Oct 5;9(4):e35882. doi: 10.2196/35882 (PMC9536768; doi:10.2196/35882)
Supplement: Multimedia Appendix 1 [file humanfactors_v9i4e35882_app1.docx]

## Multimedia Appendix 1: Search Terms

**Concept 1: Public Health**

**Keywords**: public health; community health; global health; global public health; international health; population health; healthcare; health care; health

**Concept 2: Chatbot**

**Keywords**: conversational agent; conversational bot; conversational system; conversational interface; chatbot; chat bot; chat-bot; chatterbot; dialog system; dialog agent; interactive agent; interactive virtual assistant; smartbot; smart-bot; smart bot; virtual agent; virtual coach
