# Supplementary material for: The Development and Use of Chatbots in Public Health: Scoping Review
Source: JMIR Hum Factors. 2022 Oct 5;9(4):e35882. doi: 10.2196/35882 (PMC9536768; doi:10.2196/35882)
Supplement: Multimedia Appendix 2 [file humanfactors_v9i4e35882_app2.docx]

## **Multimedia Appendix 2. Chatbot application domain, purpose, interaction type, and findings summary**

| **Citation** | **Field of use** | **Purpose of chatbot** | **Interaction modality** | **Summary of findings** |
| --- | --- | --- | --- | --- |
| Morse et al [23], 2020 | Triage | Symptom checker | single-choice selection input | Population based descriptive study of a symptom checker chatbot in the US investigating user demographics and triage acuity. 26,646 assessments were analyzed. Triage recommendation acuity was overall comparable to distribution of US-based nurse-staffed telephone triage lines from previous studies. Users were broadly representative of the regional population, but skewed towards younger people and women. |
| Ta et al [27], 2020 | Counseling / Support | Companionship | free text entry | Thematic analysis of Google Play user reviews (n=4,434) and of a user survey (n=66) with open ended questions regarding user's experiences using the chatbot. Findings suggest the benefit of artificial agents as general companions in addition to specific health interventions, but more research is necessary to investigate companion artificial agents and the potential benefits for social support, health and wellbeing. |
| Roman et al [18], 2020 | Health Literacy | Information outreach | combination of free text entry + single-choice selection input | User experience assessment (n=50) of users' perceptions and attitudes towards a chatbot about blood donation in Brazil, using a closed question survey (the User Experience Questionnaire, or UEQ) and 3 open ended questions.  The overall UX was positive. All 26 items in the UEQ scale had a mean rating over 0.8 which indicates a positive experience (for a range of -3 to 3). There were some statistically significant associations between some of the scores of the UEQ and the participants' demographic variables.  Open-ended feedback from users was positive, and justifications referred to the chatbot's content, interaction style and perceived benefits of using it. |
| Bickmore et al [38], 2020 | Health Literacy | Information outreach | single-choice selection input | 2 RCTs reported: 1) a two-armed trial to improve patient education and safety after hospital discharge to reduce rehospitalization, and 2) a two-armed trial to promote walking in older adults to improve number of steps per day. Measures assessed were health literacy, usability and attitudes towards the chatbot.  Overall, there were very few differences in measures of acceptance and usability between patients with adequate and inadequate health literacy, and where there were differences, these were mostly in favor of patients with inadequate health literacy. Participants with inadequate health literacy completed fewer conversations with the chatbot at home compared to participants with adequate health literacy (p < .05). |
| Bonnevie et al [16], 2021 | Health Promotion | Information outreach - sexual and reproductive health | free text entry | A descriptive study of chatbot co-creation through focus groups with a local community of women in the US. Only chatbot usage was reported as results: 4,390 messages received related to contraception or sexual health, and the associated campaign has showed 2,483,683 impressions, average daily reach of 1,710 for Facebook and 943 for Instagram, and 32,816 total engagements across all platforms. |
| Maeda et al [20], 2020 | Health Promotion | Information outreach - sexual and reproductive health | free text entry | A three-armed RCT was conducted online in Japan with women aged 20–34 years (n = 927) who were randomly allocated to one of three groups: a fertility education chatbot (intervention group), reading a document about fertility and preconception health (control group 1), or a document on an irrelevant topic (control group 2).  The chatbot improved fertility knowledge (+9.1 points, 15% gain, P < 0.001), but less than reading a document about the topic (+14.9 points, 24% gain, P < 0.001). As expected, no significant change was observed in control group 2. Post-test increases in the intentions to optimize behaviors were also similar between the intervention group and control group 1, and significantly higher than in control group 2. Post-test state anxiety scores were significantly lower in the intervention group than in the two control groups.  User feedback about the chatbot suggested technical limitations (e.g., low comprehension of users’ words) and pros and cons of using the chatbot (e.g., convenient versus coldness). |
| Jack et al [25], 2020 | Screening support | Information outreach - sexual and reproductive health | single-choice selection input | A two-armed RCT with 100 nongravid young women who were screened for over 100 PHC risks, and then randomly allocated to the chatbot group and the control group, who received a letter indicating their health risks and recommending a consultation with their clinician.  The chatbot reduced the number of risks over a period of 6 months more than the control group (8.3 vs. 5.5 fewer risks, P < .05, n=91).  The chatbot group averaged 63.7 minutes of interaction time. 78% reported that the chatbot was easy to use and 64% mentioned using the information from the chatbot to improve their health. |
| Fan et al [28], 2021 | Screening support | Symptom checker | combination of free text entry + single-choice selection input | Descriptive usage analysis of a self-diagnosis chatbot in China. Data consisted of 47,684 consultation sessions initiated by 16,519 users over 6 months, and included user demographic information, conversation, and user feedback.  The chatbot was used by people of all age groups, including middle-aged and older adults. The topics were diverse, including those affected by privacy and social stigma. Key issues identified were: (1) users dropping out in the middle of session (35.60%), (2) some users pretended to have health concerns and used the chatbot for non-therapeutic purposes (8.03%), and (3) user concerns about the quality of the chatbot (e.g. insufficient actionable information, perceived inaccurate diagnostic suggestions). |
| Echeazarra et al [39], 2021 | Self management | Health monitoring - blood pressure monitoring | combination of free text entry + single-choice selection input | A two-arm RCT over 2 years in Spain with 112 patients: 55 patients using a chatbot and 57 in the control group, recording measurements on paper.  The chatbot users (n=55) recorded slightly fewer BP measures than users in the control group (n=57), but the difference was not significant (mean = 12.0 vs 13.4, p = 0.109).  Chatbot users scored better in terms of knowledge and skills on BP checking best practices (mean = 24.23 vs 17.60, p = 0.03737).  92.5% of patients reported the chatbot as quite easy or very easy to use, while 3 patients who thought it was quite difficult. All respondents found it quite useful or very useful, and 85% continued to use the bot even after the experiment was over. |
| Munsch et al [22], 2020 | COVID | Symptom checker | N/A - multiple chatbots analysis | Evaluation of 10 COVID-19 symptom checkers (9 in English, 1 in German) by assessing 50 COVID-19 case reports alongside 410 non-COVID-19 control cases.  The number of correctly assessed COVID-19 and control cases varied considerably between symptom checkers, with different symptom checkers showing different strengths with respect to sensitivity and specificity. A good balance between sensitivity and specificity was only achieved by two symptom checkers. |
| McKillop et al [6], 2021 | COVID | Information outreach | N/A - multiple chatbots analysis | Analysis of usage data for 37 institutions that used Watson Assistant to develop COVID-19 chatbots.  Most institutions were governmental (n = 24), but also employers (n = 7), providers (n = 5), and health plans (n = 1). Most organizations leveraging this technology were located in the U.S. and Canada (n = 29), Europe (n = 4), and Asia Pacific (n = 4). Across all 37 organizations, over 6.8 million messages were delivered. |
| Judson et al [17], 2020 | COVID | Screening/Symptom checker | combination of free text entry + single-choice selection input | The design process of a COVID-19 chatbot to reduce screening time is described.  The screening process was shortened from a mean of 48 seconds (n=28, manual process, measured from when an employee reached the front of the line), to a mean of 8 seconds (n=20) using the chatbot.  During peak shift change, the average time waiting in line was reduced from a mean of 8 minutes 20 seconds to 1 minute 40 seconds. To date, the authors estimate that the chatbot screening tool has saved employees over 15,000 hours of time waiting to be screened. |
| Höhn & Bongard-Blanchy [8], 2021 | COVID | Symptom checker & Information outreach | N/A - multiple chatbots analysis | Study proposes a generic evaluation framework with 12 heuristics based on Nielsen’s 10 usability heuristics and adapted to the conversational interface context. 24 COVID-19 chatbots were analyzed with the proposed framework.  Identified strengths of COVID-19 chatbots include immediate feedback, familiar language, consistent wording and visual design.  Shortcomings include little user control and freedom, missing permanent menu and help options, lack of context understanding and interaction management capabilities. |
| Hautz et al [26], 2021 | COVID | Triage | single-choice selection input | A triage chatbot was developed and deployed as a non-commercial initiative in Switzerland.  During the first 40 days of the triage tool, the site saw more than 17,300 visitors, of which 69.8% indicated they would have contacted the healthcare system if the online test had not been available. Overall, since the implementation of the tool, 26.27% of all users of the site have been directed to obtain testing. The chatbot tool has had approximately 50 consults/day. |
| Lai et al [9], 2020 | COVID | Symptom checker | single-choice selection input | A triage chatbot was developed and deployed in Massachusetts General Brigham Hospital in the US to support a COVID-19 hotline manned by nurses.  The chatbot helped to triage visitors to the website and redirect only those needing further attention to the hotline clinician, thus decreasing the burden of work on staff. |
| Oh et al [29], 2020 | Mental health | Psychotherapy - CBT for panic disorder | combination of free text entry + single-choice selection input | A two-arm RCT with 41 patients randomly assigned to either a chatbot group (n = 21) or control group (n = 20) for a period of 4 weeks.  Chatbot users scored significantly lower on the Panic Disorder Severity Scale (PDSS) after using the chabot (t20 = 2.68; p = 0.01, n = 21), than the control group which were provided with a book on panic disorder (n=20).  Chatbot users also scored significantly lower on the APPQ-social phobia score and higher on the ACQ-control helplessness score compared to the control group.  There was no other difference between chabot users and the control group on the other APPQ subscales, HADS scale, BSQ scale, and ACQ subscales used in the study.  There was no significant difference in the SUS usability score between the chatbot and control groups (64.5 ± 17.0, and 69.5 ± 17.2, respectively; p = 0.35). |
| Ly et al [30], 2017 | Mental health | Psychotherapy - mental wellbeing & CBT | combination of free text entry + single-choice selection input | A two-arm RCT with 28 participants randomized to either receive the chatbot intervention (n = 14) or to a wait-list control group (n = 14).  Considering all chatbot participants, there were no significant improvements compared to the wait-list control group. However, the chatbot users who adhered to the intervention (n=13) reported improved psychological well-being (FS) (F_1, 27_ = 5.12, p = 0.032, d = 0.14); and perceived stress (PSS-10) (F_1, 27_ = 4.30, p = 0.048, d = 1.06) compared to the control group. There was no significant difference between the chatbot group and the control group in regards to life satisfaction (SWLS).  Qualitative data referred to the positives and negatives of using the chatbot. |
| Klos et al [41], 2021 | Mental health | Psychotherapy - depression & anxiety management | free text entry | A two-arm RCT with 181 Argentinian college students aged 18 to 33, randomized to either receive the chatbot intervention (n=99, only 39 reported at week 8) or assigned to a psychoeducation book on depression (n=82, only 34 reported at week 8).  The mean scores for anxiety and depressive symptoms were lower in the experimental group compared to the control group, but the difference was not statistically significant: for anxiety (t_48_= 1.74; P=.09) the effect size was moderate (d=0.5), for depression (U=448.00; P=.48) the effect size was nonexistent (d=0.09). The decrease in anxiety symptoms for the experimental group at week 8 was statistically significant (t_26_=2.15; P=.04); no such difference was observed for the control group for anxiety, or for both groups for depressive symptoms.  Feedback from most participants (25/39) at week 8 was coded as positive. A higher number of messages exchanged with the chatbot was associated with positive user feedback (F_2,36_=4.37; P=.02). |
| Fitzpatrick et al [31], 2017 | Mental health | Psychotherapy - CBT | combination of free text entry + single-choice selection input | RCT with 70 participants aged 18-28, randomized to a chatbot group (n=34 of which 31 reported data at the end of study 2 weeks later) or directed to a NIMH ebook on depression as an information-only control group (n=36 of which 25 reported data).  Chatbot group reported significantly reduced symptoms of depression as measured with PHQ-9 (F1,48=6.03; P=.017), representing a moderate between-group effect size (d=0.44). No significant between-group difference observed for anxiety or affect.  Within-group, completers experienced a significant reduction in symptoms of anxiety between baseline and at the end of study on GAD-7 (F1,54=9.24; P=.004, d=0.37), regardless of the group to which they were assigned. No effects were observed for affect as measured by the PANAS.  Qualitative data referred to the positives and negatives of using the chatbot. |
| Dosovitsky et al [42], 2020 | Mental health | Psychotherapy - depression management | free text entry | Descriptive analysis of usage data from 354 users to understand participant flow through different information modules of the chatbot.  Users engaged differently with the different modules based on length, complexity, content and style of questions within the modules, but no overall engagement patterns observed. |
| Bickmore et al [37], 2010 | Mental health | Promoting medication adherence & physical activity | single-choice selection input | 31 day quasi-experimental pilot study with 20 participants, of which 16 completed the study.  Self-reported participant dose adherence ranged from 46% to 100%, M = 89% (SD = 16%). Self-reported day adherence ranged from 8% to 100%, M = 85% (SD = 26%). Participants reported high levels of enjoyment related to their walking behavior (M = 4.5, SD = 0.31).  Ease of use ratings were highly varied in early measurements, with universally high ratings at the end, and was not correlated with system use r = −.057. Relationship with the agent ratings also had a high amount of variance among repeated measures, but was significantly correlated with system use, r = .642, p < .05. |
| Darcy et al [32], 2021 | Mental health | Psychotherapy - CBT | combination of free text entry + single-choice selection input | A retrospective study was used to analyze data from 36,070 adult users who self-referred to a CBT-based chatbot between November 2019 and August 2020.  The mean PHQ-2 score was 3.03 (SD 1.79), and 54.67% (19,719/36,070) of users scored over the cutoff score of 3 for depression screening. Within 5 days of initial app use, the mean working alliance WAI-SR score was 3.36 (SD 0.8) and the mean bond subscale score was 3.8 (SD 1.0). This is comparable to those in recent studies from the literature on traditional, outpatient, individual CBT and group CBT (mean bond subscale scores of 4 and 3.8, respectively). PHQ-2 scores at baseline correlated with bond scores (r=−0.04; P<.001). Users with depression and those without depression had high bond scores of 3.45. |
| Schroeder et al [33], 2018 | Mental health | Psychotherapy - DBT | combination of free text entry + single-choice selection input | Field study over 4 weeks with 73 participants randomized in two intervention groups, the first group receiving semi-personalised messages through the chatbot, and the second receiving non-personalised messages.  Participants significantly improved on both the PHQ-9 (B= -0.79, p<0.001) and the OASIS (B= -0.66, p<0.001) between the intake survey and week 1 of use, and continued improving (though at a slowing rate) throughout the study. On the DBT Ways of Coping Checklist (which was evaluated only at the intake and exit surveys), participants also saw an average decrease in dysfunctional coping (B= -0.06, p <0.001) and blaming others (B= -0.04, p<0.05), as well as an increase in their use of DBT skills (B= 0.04, p<0.001), from baseline to post-study. |
| Inkster et al [34], 2018 | Mental health | Supportive chatbot - mental wellbeing | combination of free text entry + single-choice selection input | A field study with 129 users categorized based on their chatbot usage into high users (n=108) and low users (n=21).  The average mood improvement between the groups revealed that the high users group had significantly higher average improvement (mean 5.84, SD 6.66) compared with the low users group (mean 3.52, SD 6.15): p=.03 and with a moderate effect size (d=0.63). 67.7% of user-provided feedback responses found the app experience helpful and encouraging. |
| Xu & Zhuang [21], 2020 | Mental health | Psychotherapy | free text entry | Comparative analysis of chatbots and respective evaluation studies indicating that chatbots are beneficial for some people with mental health conditions. |
| Greer et al [43], 2019 | Health Monitoring | Supporting mental wellbeing | combination of free text entry + single-choice selection input | A two-armed RCT with 45 participants over 8 weeks, randomly assigned into the intervention group which received access to the chatbot immediately, and the control group which received access to the chatbot after 4 weeks.  After 4 weeks, the intervention group reported an average reduction in anxiety compared with the control group (intervention reduction of 2.58 standardized t-score units vs control increase of 0.7 units, p=.09, d=-0.41). Those in the intervention group also experienced greater reductions in anxiety when they engaged in more sessions (z=–1.9, p=.06). There were no significant differences in depression, positive emotion, or negative emotion for either group.  Participants in the intervention group rated their experience as helpful (mean 2.0/3, SD 0.72) and would recommend it to a friend (mean 6.9/10; SD 2.6). Open-ended feedback noted the chatbot's nonjudgmental nature as a particular benefit. |
| Kowatsch et al [45], 2021 | Treatment support | Teaching skills, Supportive chatbot - children with asthma | combination of free text entry + single-choice selection input | A single-arm feasibility study over 4 weeks with 49 participants, 37 of which finished the study.  Technology acceptance was positive for all relevant stakeholders, and there was a strong patient-conversational agent working alliance. Intervention completion rate was 75.5%. Family members supported the patients in 269 out of 275 (97.8%) coaching sessions.  Asthma knowledge scores show a significant increase and large effects (n=37, t_36_=–3.68, p<.001, d=1.19).  Qualitative feedback referenced positively the content of the chatbot, and several suggestions for improvement were made. |
| Chaix et al [35], 2019 | Treatment support | Information outreach - alleviate fears/concerns about breast cancer | combination of free text entry + single-choice selection input | A prospective study analyzing data from 4,737 patients using the chatbot.  An average of 132,970 messages exchanged per month was observed between the patients and the chatbot (Vik).  The average compliance of patients using the medication reminder feature increased over 5 weeks by more than 20% (n=33, P=.04).  In responding to the satisfaction survey questions asked by the chatbot, patients regularly left positive comments. The overall satisfaction reported to the chatbot was 93.95% (900/958). |
| So et al [40], 2020 | Behavioral change | Supportive chatbot - stopping gambling | free text entry | A two-armed RCT with 197 subjects, randomly allocated to the daily chatbot intervention group and the control group receiving only bi-weekly assessments.  There was no significant between-group difference in the primary outcome (PGSI difference - 1.14, p = 0.162, effect size of d=0.4) but in the secondary outcome the G-SAS was significantly lower in the intervention group than in the control group (difference - p = 0.03). |
| Gardiner et al [24], 2020 | Behavioral change | Information outreach - preconceptions on health risks | single-choice selection input | A two-armed RCT with 480 African American and Black women who reported at least one nutrition risk, and then randomly allocated to the chatbot group and the control group.  After 6 months, the intervention group reported progressing forward on the stage of change scale for, on average, 52.9% (SD, 35.1%) of nutrition and supplement risks compared to 42.9% (SD, 35.4) in the control group (p = 0.019), and (b) reported achieving the action and maintenance stage of change for, on average, 52.8% (SD 37.1) of the nutrition and supplement risks compared to 42.8% (SD, 37.9) in the control group (p = 0.004). For subjects beginning the study at the contemplation stage of change, intervention subjects reported progressing forward on the stage of change scale for 75.0% (SD, 36.3%) of their health risks compared to 52.1% (SD, 47.1%) in the control group (p = 0.006). |
| Maher et al [44], 2020 | Behavioral change | Coach - healthy lifestyle | free text entry | A single arm pre-post study with 81 participants aged 45 to 75 years in Adelaide Australia.  Participants completed a mean of 109.8 (95% CI 1.9-217.7) more minutes of physical activity at week 12 compared with baseline. Mediterranean diet scores increased from a mean of 3.8/14 at baseline, to 9.6/14 at 12 weeks (mean improvement 5.7 points, 95% CI 4.2-7.3). After 12 weeks, participants lost an average 1.3 kg (95% CI -0.1 to -2.5 kg) and 2.1 cm from their waist circumference (95% CI -3.5 to -0.7 cm). There were no significant changes in blood pressure. Feasibility was excellent in terms of recruitment, retention (90% at 12 weeks), and safety (no adverse events). |
| Perski et al [36], 2019 | Behavioral change | Supporting chatbot - smoking cessation | single-choice selection input | A two-armed RCT trial with 57,214 smokers (intervention: 9.3% (5339); control: 90.7% (51,875)) tested the inclusion of a chatbot in a smoking cessation application.  The intervention led to a 101% increase in engagement (p < .001).  Smokers allocated to the intervention had greater odds of quit success at 1 month (statistically significant considering both all participants and only completers, p < .001), but due to a low follow-up rate of 10.6% (intervention: 19.9%, control: 9.7%), this is low quality evidence that the chatbot also increased self-reported smoking cessation. |
